# Applications of neuronavigation system in cranial surgery: experience of a single center

**Published:** 2012-11

**Authors:** Mohsen Nouri, Mehrdad Pahlavani, Abbas Amirjamshidi, Mohammad Shirani-Bidabadi, Ebrahim Ketabchi, Kourosh Karimi-Yarandi

**Affiliations:** ^*a*^Department of Neurosurgery, Sina Hospital, Tehran, Iran.

## Abstract

**Background:**

Since the advent of navigational systems in neurosurgery, various implications have been introduced for them in spine and brain practices. Although, the range of surgeries in which these systems are being used is getting wider over time, their application is becoming more specific in certain situations.

**Methods:**

This means that defining specific indications for their usage is not as easy as it was previously thought. Brief reviewing of the available literature showed various navigation systems proposed and used in neurosurgery.

**Results:**

In this study, we review case selection criteria, techniques, and the results of neuronavigation applications in the patients underwent neurosurgical operations in the Sina hospital (Tehran, Iran) during 2011.

**Conclusions:**

The findings of our survey and experiences prove the efficacy and advantages of this technique that reduces the risk of neurovascular damage, neural tissue manipulation, operation time, and bleeding.

**Keywords:**

Neuronavigation, Cranial surgery, Navigational system


					Volume 4, Suppl. 1 Nov 2012
Publisher: Kermanshah University of Medical Sciences
URL: http://www.jivresearch.org
Copyright:
Congress of Iranian Neurosurgeons, 14-16 November 2012, Kermanshah, Iran
Paper No. 19
Applications of neuronavigation system in cranial surgery:
experience of a single center
Mohsen Nouri a
, Mehrdad Pahlavani a
, Abbas Amirjamshidi a,*
, Mohammad Shirani-Bidabadi a
,
Ebrahim Ketabchi a
, Kourosh Karimi-Yarandi a
a
Department of Neurosurgery, Sina Hospital, Tehran, Iran.
Abstract:
Background: Since the advent of navigational systems in neurosurgery, various implications have been introduced for
them in spine and brain practices. Although, the range of surgeries in which these systems are being used is getting
wider over time, their application is becoming more specific in certain situations.
Methods: This means that defining specific indications for their usage is not as easy as it was previously thought. Brief
reviewing of the available literature showed various navigation systems proposed and used in neurosurgery.
Results: In this study, we review case selection criteria, techniques, and the results of neuronavigation applications in
the patients underwent neurosurgical operations in the Sina hospital (Tehran, Iran) during 2011.
Conclusions: The findings of our survey and experiences prove the efficacy and advantages of this technique that
reduces the risk of neurovascular damage, neural tissue manipulation, operation time, and bleeding.
Keywords:
Neuronavigation, Cranial surgery, Navigational system
* Corresponding Author at:
Abbas Amirjamshidi: Department of Neurosurgery,Sina Hospital,Tehran, Iran,Tel:09121329864, Email:abamirjamshidi@yahoo.com, (Amirjamshidi A.).

				

